# Supplementary material for: Porous carbon nanowire array for surface-enhanced Raman spectroscopy
Source: Nat Commun. 2020 Sep 24;11:4772. doi: 10.1038/s41467-020-18590-7 (PMC7519110; doi:10.1038/s41467-020-18590-7)
Supplement: Supplementary file 1 — Supplementary Information [file 41467_2020_18590_MOESM1_ESM.pdf]

Supplementary Information

for

Porous carbon nanowire array for surface-enhanced Raman spectroscopy

by Chen et al.

Supplementary Note 1: Comparison with different types of SERS substrates

We compare different types of SERS substrates, namely, Au, Ag, MoS_2, Al_xO_3, and graphene in terms of photothermal heat generation, reproducibility, biocompatibility, and enhancement factor, as shown in Supplementary Table 1. For the metal substrates (Au and Ag), they provide high enhancement factors that depend on hot spots, but suffer from large photothermal heat generation, seriously limiting their reproducibility. For the semiconductor nanostructures and metal oxides (MoS_2 and Al_xO_3), their enhancement factors are on the order of 10^4-10^5. Due to the inherent photocatalytic activity and the toxicity of the substrate materials to biomolecules, the semiconductor nanostructures suffer from poor biocompatibility. On the other hand, as a carbon allotrope, graphene exhibits excellent biocompatibility. Moreover, due to the fluorescence-quenching effect of graphene, it has been used in conjunction with other metal nanostructures for SERS measurements, which is known as graphene-enhanced Raman scattering (GERS). However, the enhancement factor of GERS is normally moderate (~10^3) compared with the pure metal substrates. Among the previous substrates, the PCNA exhibits a high enhancement factor (~10^6) with low heat generation, high reproducibility, and high biocompatibility.

Supplementary Note 2: Experimental materials

PPy was purchased from Aladdin Bio-Chem Technology Co. Ltd., China. AAO templates (Anodisc 13, Cat. No. 6809-7013) with a thickness of 60 μm, a diameter of 13 mm, and an interior porous diameter of 100 nm were purchased from Whatman Co. Ltd., USA. An SEM image of the AAO with a porosity of 50-60% is shown in Supplementary Fig. 1. All the chemicals in this study were purchased from Sigma-Aldrich Co. Inc., China, and used without modification. The commercial metal substrates (the Ag-Au nanoparticle hybrid substrates) were produced by SERSitive Co..

Supplementary Note 3: Growth of the PCNA

A conductive layer was obtained by sputtering a 1-μm-thick Au layer on one side of the AAO template before the electropolymerization process. A home-made three-electrode test system, which is composed of an AAO template as the working electrode, a silver/silver chloride electrode as the reference electrode, and a platinum (Pt) foil as the counter electrode, was used to deposit the PNA into the AAO template. During the deposition process, we used a cyclic voltammetry (CV) method at a scan rate of 500 mV/s in a chromatographically pure acetonitrile solution containing 0.1 M LiClO4 and 0.1 M pyrrole. The electrochemical deposition was performed

in a home-made electrochemical cell at room temperature. The electric potential range of the CV method was controlled within 0-1.0 V. After the electrodeposition process, the sample was eluted in deionized water several times. Then, the PNA was treated by the electro-degradation process by using a 100 °C dimethyl sulfoxide (DMSO) solution with suspended sulfur clusters. Nanopores in the PNA were formed by using the CV method at a scan rate of 200 mV/s. Finally, the PPNA was annealed at a temperature of 800 °C for 2 h under an argon atmosphere at a heating rate of 10 °C/min to obtain a PCNA substrate.

Supplementary Note 4: Fabrication of the PCNA substrate

The Au layer on the bottom side of the AAO template was first removed by using a mechanical polishing method. Then, the sample was immersed in aqua regia to remove the remaining Au before transferring the PCNA to a bare Si wafer. After that, the AAO template on the Si wafer was removed by using an aqueous NaOH solution with a concentration of 6 M. Since the bottom of the PCNA became interconnected by PPy during the electropolymerization process, the PCNA remained intact after the removal of the Au and AAO template. After being washed in deionized water, the PCNA substrate on the Si wafer was finally obtained. Several PCNA substrates were prepared by using the same recipe. All the PCNA substrates were kept in a sealed and dry environment before characterizations and SERS measurements. Supplementary Fig. 3 shows a black PCNA substrate placed on a white poly tetrafluoroethylene (PTFE) plate.

The height and diameter of the PCNA substrate can be controlled by changing the AAO template dimensions. For the pore size and pore density, they are mainly controlled in the electro-degradation step. As the pore size and porosity are highly dependent on the electro-degradation time and rate, we can control the pore size and the porosity by tuning the electro-degradation time and rate. The electro-degradation rate is tunable by varying the current density of electro-degradation as well as the pH value and concentration of the DMSO solution.

Supplementary Note 5: Thermogravimetric analysis of the PNA

Thermogravimetric analysis (TGA) of the PNA was conducted by using a thermobalance (TGA 2050). In the analysis, the PNA was placed in an Argon atmosphere where the temperature was increased from 20 °C to 800 °C at a rate of 10 °C/min. We found that the PNA began to lose its composition ratio quickly and became carbonized at a temperature of 500 °C, as shown in Supplementary Fig. 2. Therefore, in order to carbonize the PNA sufficiently, we chose 800 °C.

Supplementary Note 6: Optical properties of the PCNA

We conducted a numerical study of the optical properties of the PCNA nanowires with differential physical dimensions as shown in Supplementary Fig. 4. No structural resonance peaks are evident in the calculated reflection and absorption spectra as the diameters of the PCNA nanowires are much smaller than the excitation wavelength, which is also an additional piece of evidence for the low electromagnetic enhancement. The physical

dimensions of the PCNA nanowires are mainly selected to optimize the chemical enhancement as the electromagnetic enhancement contributes little to the overall Raman signal enhancement due to the absence of localized surface plasmon resonance (LSPR). First, we designed a well-aligned 2D array as a SERS substrate to enable high spatial reproducibility for SERS measurements. Second, we selected long porous nanowires as 3D inclusions of the array to enhance the adsorption of detected molecules on the PCNA substrate and the molecule-PCNA interaction area for chemical enhancement. We chose the length of the nanowires to be as long as 15 μm (much larger than the size of the focused light spot) to provide a sufficiently large 3D region for a highly efficient Raman enhancement. Third, we selected the nanowires with small diameters to increase the specific surface area. We chose the diameter of the nanowires to be 140 nm to enable not only a large specific surface area, but also excellent mechanical stability for maintaining the structure of a well-aligned array.

Supplementary Note 7: SEM imaging of PNA, CNA, and PCNA substrates

SEM images of the PNA, CNA, and PCNA substrates, taken from a JSM-7600F FESEM microscope, were compared in Supplementary Fig. 5a and Supplementary Fig. 5b. These figures clearly show that the electrolysis and carbonization processes had little influence on the surface flatness of the nanowires, respectively. Moreover, the PCNA maintained the original one-dimensional morphology and possess an average diameter of 140 nm after the carbonization process, as shown in Supplementary Fig. 5c.

Supplementary Note 8: Nitrogen adsorption-desorption isotherm measurements of the PNA and PCNA substrates

The SSAs were determined by conducting nitrogen adsorption-desorption isotherm measurements at a temperature of 77 K (NOVA 2200e). As shown in Supplementary Fig. 6a, the hysteresis loop of the PCNA substrate is larger than that of the PNA at low pressure, indicating an increased number of pores. The Brunauer-Emmett-Teller (BET) SSAs of the PCNA was calculated to be 547.13 m^2/g, which is higher than that of the PNA (363.63 m^2/g), due to the presence of more nanopores in the PCNA. Moreover, a large number of pores on the nanowires further increased the surface roughness of the PCNA substrate, as shown in Supplementary Fig. 6b, which contributed to higher enhancement in SERS.

Supplementary Note 9: Methods for characterizing the PPNA and PCNA substrates

The morphology and structure of samples (substrates) were characterized by using an SEM (JEOL JSM-7600F) at an accelerating voltage of 5 kV. An energy dispersive X-ray (EDX) detector equipped with the SEM was used to characterize the surface morphology and element composition of the sample at an accelerating voltage of 15 kV. The current-voltage (I-V) characteristics of the PPNA and PCNA substrates were recorded with a Keithley 4200 at room temperature (25 °C) in the air, as shown in Supplementary Fig. 7. The specific surface areas (SSAs) were measured by using a nitrogen adsorption-desorption isotherm method at a temperature of 77 K (NOVA 2200e). The Raman spectra of the PPNA and PCNA substrates were obtained by using an RM 2000 microscopic

confocal Raman spectrometer (Renishaw PLC, England) excited by a 514-nm-wavelength continuous-wave laser.

Supplementary Note 10: X-ray diffraction measurements of the PCNA

X-ray diffraction (XRD) analysis was performed on the PCNA with a D8 Advance X-ray diffractometer with CuKα radiation (λ = 1.5418 Å) in the range of 5-80° (2θ) with a scanning step of 0.05° at a temperature of 25 °C. As shown in Supplementary Fig. 8, the PCNA has two diffraction peaks at 26° and 44°, which correspond to the characteristic peaks of graphite, specifically the (002) and (100) crystal planes of the graphite structure[1]. The results showed that the PPNA was successfully carbonized at a temperature of 800 °C to produce partially graphitized carbon.

Supplementary Note 11: Raman peak assignments of R6G, β-lactoglobulin, glucose, and DMSO

Our Raman peak assignments of R6G, β-lactoglobulin, and glucose are shown in Supplementary Fig. 9a through Supplementary Fig. 9d. For R6G, Supplementary Fig. 9a shows its Raman spectrum on the PCNA substrate after a 30-s incubation in a 10-μM R6G solution. A detailed assignment of the R6G spectral features has been reported previous[2], which is highly consistent with our results. In the spectral region between 1000 and 1700 cm^{-1}, a strong band is evident due to the stretching mode of the carbon skeleton. The stretching modes of the carbon skeleton give rise to the strongest R–R band. Moreover, the C–C stretching modes of R6G result in strong bands at 1185, 1309, 1361, 1507, 1575 and 1650 cm^{-1} in the spectrum. For β-lactoglobulin, its Raman spectra in the spectral range of 900-1600 cm^{-1} with a mass fraction of 0.4% on the silicon and PCNA substrates are shown in Supplementary Fig. 9b. The Raman peak at 1001 cm^{-1} is due to the C–C ring stretching vibrations of phenylalanine (Phe), which is usually separated from other bands and its intensity is not affected by protein conformational changes. The Raman peak at 1245 cm^{-1} is the amide III mode region, which is mainly produced by C–N stretching and N–H in-plane bending vibrations of peptide bonds. The bending and wagging modes of C–H of β-lactoglobulin appear at 1376 and 1454 cm^{-1}. The Raman peak at 1547 cm^{-1} can be assigned to the changes in disulfide conformation as well as in microenvironment around amino acid residues[3,4]. For glucose, its Raman spectra in the spectral range of 800-1600 cm^{-1} with a mass fraction of 0.1% on the silicon and PCNA substrates are shown in Supplementary Fig. 9c. The Raman peak at 836 cm^{-1} can be assigned to the stretching mode of C–C. The Raman peak at 924 cm^{-1} can be assigned to the bending mode of C–H. The Raman peak at 1028 cm^{-1} can be assigned to the stretching mode of C–O. The Raman peak at 1255 cm^{-1} can be assigned to the twisting mode of CH_2, while the Raman peaks at 1350 cm^{-1} can be assigned to the wagging mode of CH_2. The Raman peak at 1403 and 1480 cm^{-1} can be assigned to the bending mode of CH_2[5]. For DMSO, the Raman spectra in the spectral range of 500-1500 cm^{-1} with a mass fraction of 5.5×10^{-8}% on the silicon and PCNA substrates are shown in Supplementary Fig. 9d. The C–S stretching mode results in the Raman peaks at 681 and 718 cm^{-1}, while the bending mode of C–H appears at 949 cm^{-1}. The Raman peaks at 1010 cm^{-1} can be assigned to the stretching mode of S=O. The Raman peak at 1311 cm^{-1} can be assigned to the C–H symmetric deformation.

Moreover, we attribute the Raman peak at 1419 cm^{-1} to the CH_3 degenerate deformation[6-8]. The peak assignments of observed Raman shifts of R6G, β-lactoglobulin, glucose, and DMSO are summarized in Supplementary Table 2.

Supplementary Note 12: Fluorescence quenching of the PCNA substrate

The PCNA substrate has an excellent property of fluorescence quenching. As shown in Fig. 2 and Supplementary Fig. 10, the fluorescence of R6G was significantly surppressed at the excitation wavelengths of both 532 nm and 785 nm. It is worthwhile to note that the fluorescence excited at 785 nm is the so-called anti-Stokes fluorescence in which the excitation photon energy does not need to be precisely identical to the energy gap between the ground state and an excited electronic state of the molecule such that the excitation photon energy can be smaller than the energy gap due to the thermal excitation (Boltzmann distribution). The energy difference between the photon energy and the energy gap only affects the excitation probability that determines the fluorescence intensity. A larger energy difference means a lower excitation probability which corresponds to a lower fluorescence intensity. Therefore, at the excitation wavelength of 785 nm, R6G emits fluorescence as shown in Supplementary Fig. 10, but its fluorescence intensity is much weaker (comparable to the spontaneous Raman scattering intensity as also shown in the same figure) than that at the excitation wavelength of 532 nm due to the larger energy difference.

Supplementary Note 13: Raman spectroscopy of DMSO on the silicon and PCNA substrates

To demonstrate trace-amount detection of molecules on the PCNA substrate, we conducted SERS of DMSO, a well-known polar aprotic solvent that dissolves both polar and nonpolar compounds and is miscible in a wide range of organic solvents as well as water. Supplementary Fig. 11 shows that the Raman spectra of DMSO solutions on the silicon and PCNA substrates under light excitation at 785 nm. The Raman spectrum of the DMSO solution with a mass fraction of 100% on the silicon substrate was first measured as a ground truth. On the PCNA substrate, all the characteristic Raman peaks of the DMSO solution were identified and distinguished even at a mass fraction of 5.5×10^{-8}%, corresponding to an enhancement factor of $\sim10^{7}$. At a further reduced mass fraction of 5.5×10^{-9}%, no characteristic Raman peaks of the molecules were visible. More importantly, even with such a high signal enhancement, all the measured characteristic Raman peaks of the DMSO solutions agree well with those of the ground truth. No distortion or undesirable shift of the Raman peaks was identified in the measured SERS spectra at the different mass fractions, which is attributed to the excellent sensitivity, biocompatibility, and photothermal stability of the PCNA substrate.

Supplementary Note 14: Calculation of the Raman peak intensities of R6G

A baseline correction method was chosen to minimize the effect of fluorescence on the Raman spectrum. In addition, we used a 785-nm-wavelength laser as an excitation light source to minimize the fluorescence of R6G. We measured the relative height of the peak intensity to the baseline. The differences in the relative intensities

of the Raman peaks at 1185, 1309, 1361, 1507, and 1650 cm^{-1} between all the substrates are with a standard deviation (SD) as follows:

$$\mathrm{SD} = \sqrt{\frac{1}{N-1}\sum_{1}^{N}(x_i - \bar{x})^2}, \quad (1)$$

where x_i is the relative intensity of each Raman peak, $\bar{x}$ is the average peak intensity of 20 samples at a certain Raman shift. After calculations, we found that the SD of the peak intensity at 1361 cm^{-1} (SD_{1361}) is 5.647, SD_{1185} is 5.537, SD_{1309} is 5.593, SD_{1507} is 5.540, SD_{1650} is 5.559, respectively. The SD of all the peak intensities is 5.686.

Supplementary Note 15: Estimation of the numbers of molecules of the measured samples in the probed volume

Below we estimate the number of molecules of the measured samples in the probed volume and summarize them in Supplementary Table 3. In our SERS experiments, we used a 50x objective lens with a numerical aperture (NA) of 0.42 to focus the 785-nm incident light onto the samples. Assuming the Airy disc, the probed volume can be approximately calculated by $(0.61\lambda/\mathrm{NA})^3$, where λ is the wavelength of the incident light. From our experimental values, the probed volume is found to be 1.5 μm^3.

In the SERS measurement of β-lactoglobulin, we used both β-lactoglobulin powder and solution as the samples. The density of the β-lactoglobulin powder is 1.25 g/cm^3 while its molar mass is ~18,400 g/mol[9]. Thus, its molar concentration is found to be (1.25 g/cm^3) / (18,400 g/mol) = 0.068 M. The total number of molecules of the β-lactoglobulin powder in the probed volume is found to be 0.068 M × 1.5 μm^3 = 1.0×10^{-16} mol. As the density of the β-lactoglobulin solution with a Mf of 0.4% is between 1 g/cm^3 and 1.25 g/cm^3, which is approximately identical to that of the β-lactoglobulin powder, the ratio of the number of molecules of the β-lactoglobulin solution to that of the β-lactoglobulin powder in the probed volume is approximately equal to their Mf ratio. The total number of molecules of the β-lactoglobulin solution in the probed volume is found to be 1.0×10^{-16} × 0.4% / 100% = 4.0×10^{-19} mol. Therefore, the Mf ratio approximately equals the ratio of the numbers of molecules under enhanced and unenhanced conditions, which is used to calculate the SERS enhancement factor of β-lactoglobulin.

Similarly, in the SERS measurement of glucose, the density and molar mass of the glucose powder are given by 1.56 g/cm^3 and 180 g/mol, respectively. Thus, its molar concentration is found to be 8.7 M. The total number of molecules of the glucose powder in the probed volume is found to be 8.7 M × 1.5 μm^3 = 1.3×10^{-14} mol. As the density of the glucose solution with a Mf of 0.1% is between 1 g/cm^3 and 1.56 g/cm^3, which is approximately identical to that of the glucose powder, the ratio of the number of molecules of the glucose solution to that of glucose powder in the probed volume is approximately equal to their Mf ratio. The total number of molecules of the glucose solution in the probed volume is found to be 1.3×10^{-14} × 0.1% / 100% = 1.3×10^{-17} mol. Therefore, the Mf ratio that approximately equals the ratio of the numbers of molecules under enhanced and unenhanced conditions is used to calculate the SERS enhancement factor of glucose.

Finally, in the SERS measurement of DMSO, the density and molar mass of pure DMSO are 1.1 g/cm^3 and 78 g/mol, respectively. Thus, its molar concentration is 14.1 M. The total number of molecules of the pure DMSO in the probed volume is found to be 14.1 M × 1.5 μm^3 = 2.1×10^{-14} mol. As the density of the DMSO solution with a Mf of 5.5×10^{-8}% is between 1 g/cm^3 and 1.1 g/cm^3, which is approximately identical to that of pure DMSO, the ratio of the number of molecules of the DMSO solution to that of the pure DMSO in the probed volume is approximately equal to their Mf ratio. The total number of molecules of the DMSO solution in the probed volume is 2.1×10^{-14} × 5.5×10^{-8}% / 100% = 1.2×10^{-23} mol. Therefore, the Mf ratio that approximately equals the ratio of the numbers of molecules under enhanced and unenhanced conditions is used to calculate the SERS enhancement factor of DMSO.

Supplementary Note 16: Reproducibility testing of the metal substrate in space

To quantitatively compare the metal and PCNA substrates in terms of reproducibility in space, we performed a similar spot-to-spot consistency assessment of a commercial metal substrate. Specifically, we conducted SERS mapping of the metal substrate at two characteristic Raman peaks of β-lactoglobulin (955 cm^{-1} and 1409 cm^{-1}) on both large and small scales, as shown in Supplementary Fig. 14a. The figure shows that there is a significant level of surface inhomogeneity in the enhancement factor, including the existence of position-dependent hot spots. As shown in Supplementary Fig. 14b, the Raman signal intensities measured on the metal substrate has a CV value of more than 25.3% on average, which is much larger than that of the PCNA substrate.

Supplementary Note 17: Reproducibility testing of the metal substrate in time

To quantitatively compare the metal and PCNA substrates in terms of reproducibility in time, we performed a time-to-time consistency assessment of the commercial metal substrate. The experimental conditions for this control experiment are identical to those for the PCNA substrate. As shown in Supplementary Fig. 15, several weak Raman peaks of glucose on the commercial metal substrate appeared at t = 0 h. However, no Raman peaks of the glucose molecules on the commercial metal substrate appeared at t = 1 h, 2 h, and 3 h. The results indicate that the metal substrate has a poor time-to-time consistency for SERS measurements. To quantify the temporal Raman intensity fluctuations, we determined the CV of the intensity of each characteristic Raman peak as shown in Supplementary Fig. 16, which indicates high temporal stability as evidenced by a small CV value of 15.1% on average within 4 hours as opposed to most metal SERS substrates that cannot be used for more than 1 hour as they are easily oxidized in the air.

Supplementary Note 18: Theoretical analysis of the chemical mechanism

To verify that the chemical mechanism is the dominant factor of the Raman enhancement on the PCNA substrate, we theoretically analyzed the charge-transfer mechanism using density functional theory with Gaussian16. Here we approximated the surface of the PCNA as a carbon sheet composed of six carbon membered rings and sixteen hydrogen atoms ($C_{42}H_{16}$). Our theoretical calculation models are illustrated in Supplementary Fig. 17a, where

an isolated R6G molecule and an R6G molecule on the surface of the PCNA are considered based on time-dependent density functional theory at the level of B3LYP/6-31G(d) and B3LYP/3-21G for the R6G molecule and the R6G molecule on the surface of the PCNA, respectively. First, we calculated the excitation energies required for the R6G molecule and the R6G molecule on the surface of the PCNA for resonance Raman scattering as shown in Supplementary Fig. 17b. With the assistance of the PCNA surface, the required excitation energies are significantly lowered. In particular, the two lowest emerging excitation energies (1.6 eV and 2.3 eV) enable the R6G molecule on the surface of the PCNA to be resonantly excited at the wavelengths of 785 nm (1.58 eV) and 532 nm (2.33 eV). On the contrary, the R6G molecule alone cannot be resonantly excited at the wavelength of 785 nm due to the absence of excited states between the HOMO and the lowest unoccupied molecular orbital (LUMO) and can be resonantly excited at the wavelength of 532 nm, but with a very strong fluorescence background that obscures the Raman spectrum. This difference results in the large chemical enhancement of the PCNA observed in our experiments (Fig. 4b). Based on the excitation energies calculated above, we further calculated the molecular orbitals of the R6G molecule on the surface of the PCNA to clarify its charge-transfer directions. Finally, by combining the estimation of the excitation energies and the clarification of charge-transfer directions, the charge-transfer pathways for the experimentally observed Raman enhancement of R6G on the PCNA (Fig. 4b) are also shown in Fig. 4a.

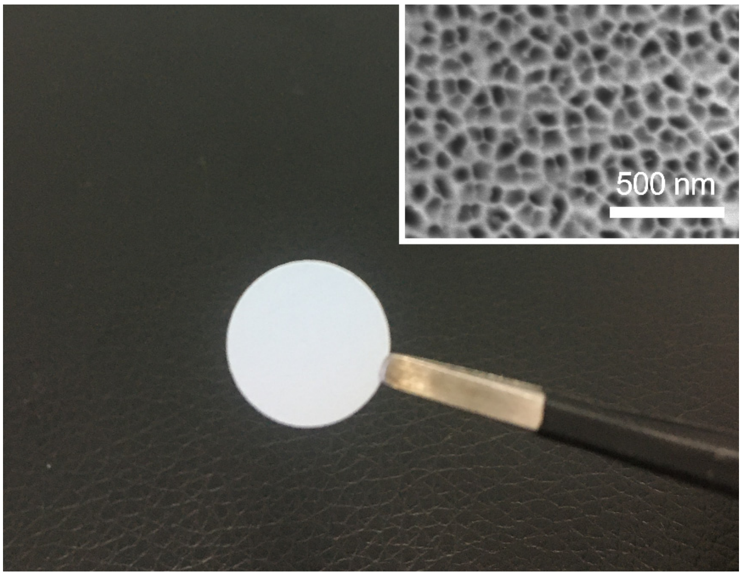

Supplementary Fig. 1 | Photo and SEM image of the AAO template with a porosity of 50-60%. The diameter of the AAO template product is 13 mm. The pores in the AAO template have a diameter of between about 100 and 150 nm as shown in the inset of the figure.

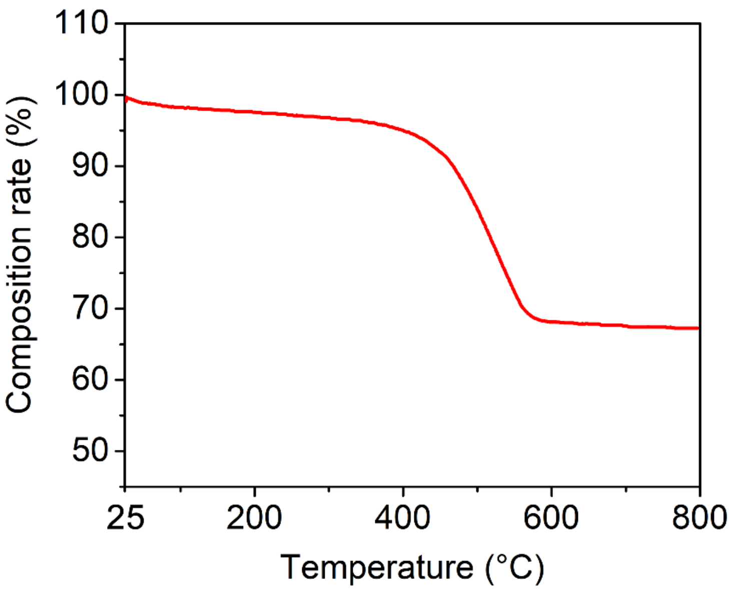

Supplementary Fig. 2 | Thermogravimetric analysis of the PNA. The measurement shows that the PNA began to lose its PPy ratio and became carbonized at a temperature of 500 °C. In addition, the sample has no significant weight loss above 600 °C. In order to carbonize the PNA, we chose 800 °C.

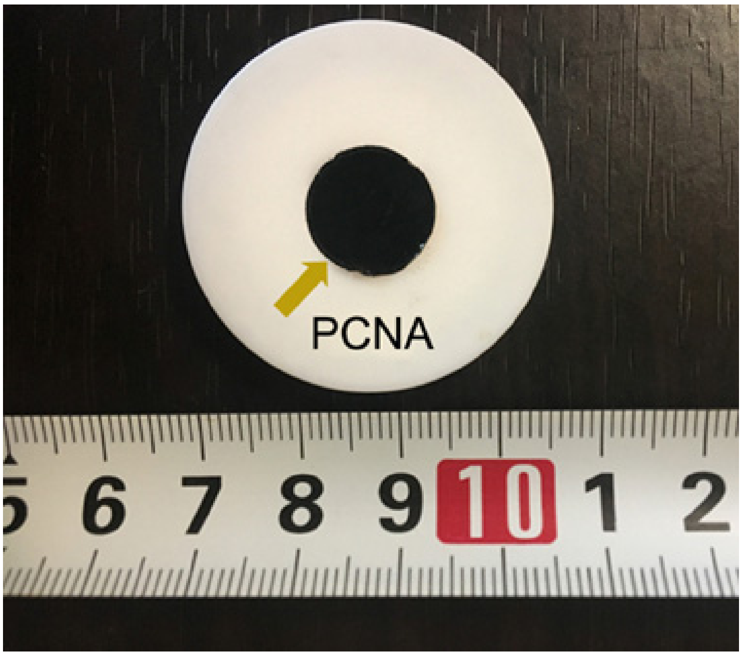

Supplementary Fig. 3 | Picture of the PCNA substrate on the PTFE plate. It is evident from the picture that the PCNA substrate has a diameter of about 12 mm.

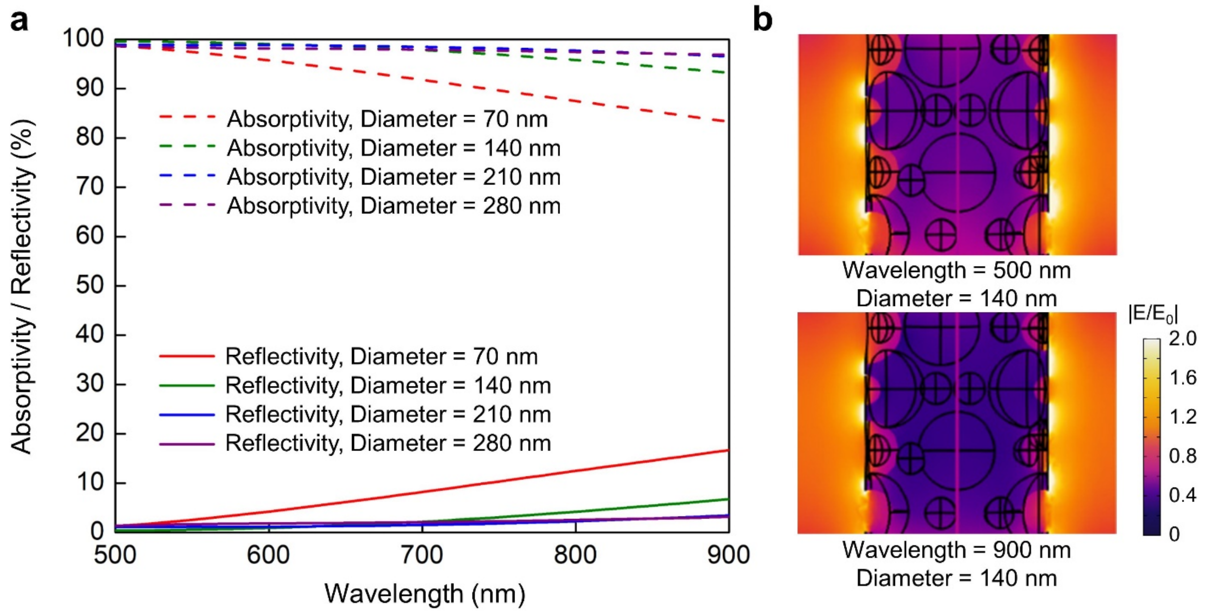

Supplementary Fig. 4 | Theoretical analysis of the PCNA substrate. a, Calculated reflection (solid lines) and absorption (dash lines) spectra of PCNA substrates comprised of porous carbon nanowires with different diameters. **b,** Electric field magnitude distribution of a porous carbon nanowire with a diameter of 140 nm at excitation wavelengths of 500 nm and 900 nm.

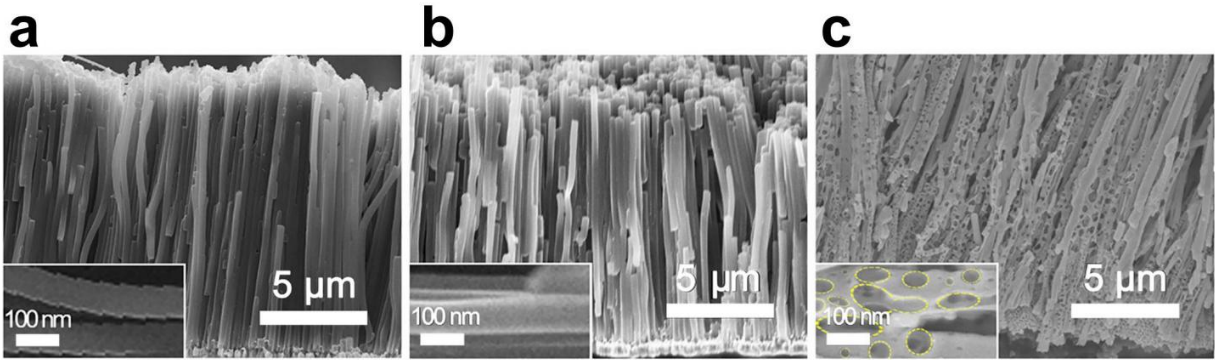

Supplementary Fig. 5 | a, PNA substrate. b, CNA substrate. c, PCNA substrate. The insets show enlarged images of the PNA, CNA, and PCNA substrates. In the inset of (c), the yellow dotted circles indicate nanopores on the PCNA.

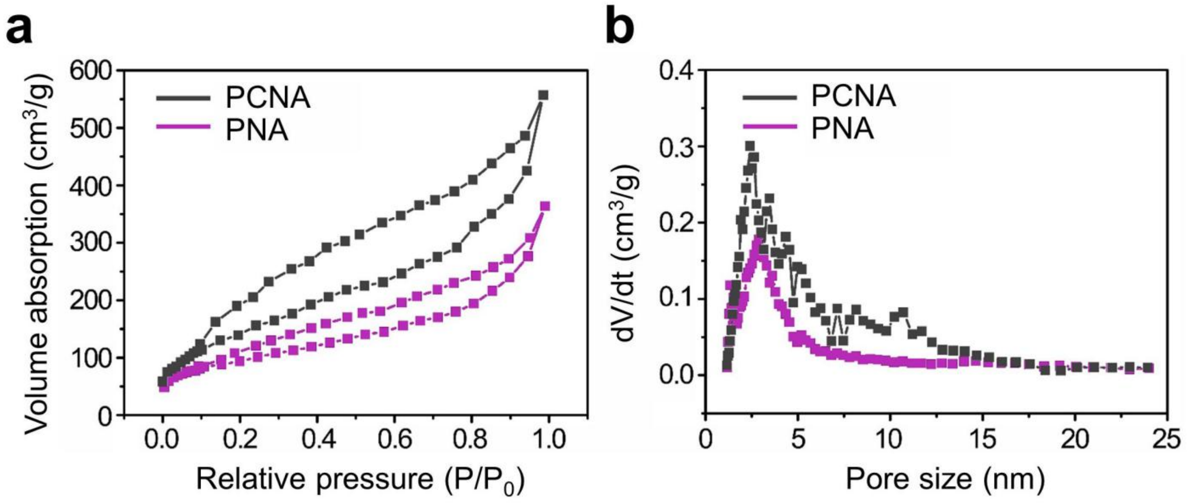

Supplementary Fig. 6 | Nitrogen adsorption-desorption isotherm measurements of the PNA and PCNA substrates. **a**, N_2 adsorption-desorption isotherms of the PNA and PCNA substrates. The hysteresis loop of the PCNA substrate is larger than that of the PNA substrate, which indicates the number of pores increased in the substrate. **b**, Pore size distributions of the PNA and PCNA substrates.

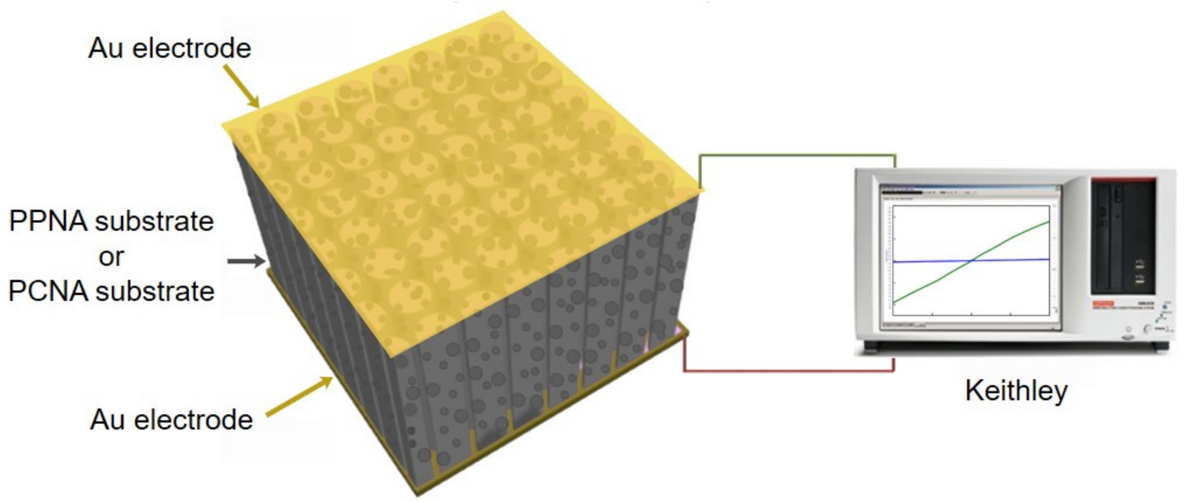

Supplementary Fig. 7 | Schematic of the *I-V* measurement setup of the PPNA and PCNA substrates. The PCNA substrate with an effective area of about 0.5 × 0.5 mm^2 was put on an Au sheet electrode and covered with another Au sheet electrode on the top of the PCNA substrate to ensure that the two Au sheet electrodes were separated. All the substrates in our experiments were developed in a top-contact device configuration. The *I-V* characteristics of the substrates were recorded with a Keithley 4200 at room temperature in air.

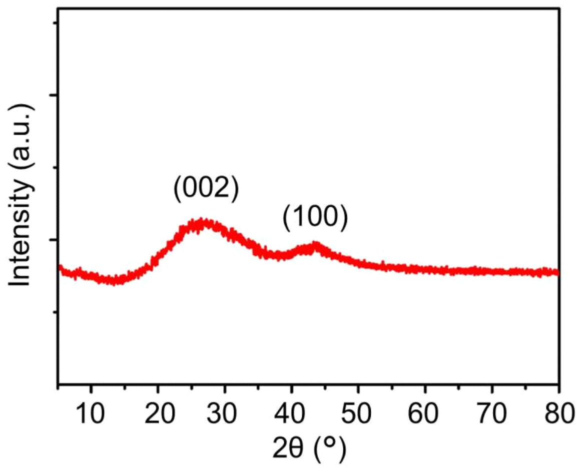

Supplementary Fig. 8 | XRD spectrum of the PCNA substrate. There are two diffraction peaks at 26° and 44° in the XRD spectrum, which correspond to the characteristic peaks of graphite. The results indicate the PPNA partially transformed to graphitized carbon after the carbonization process.

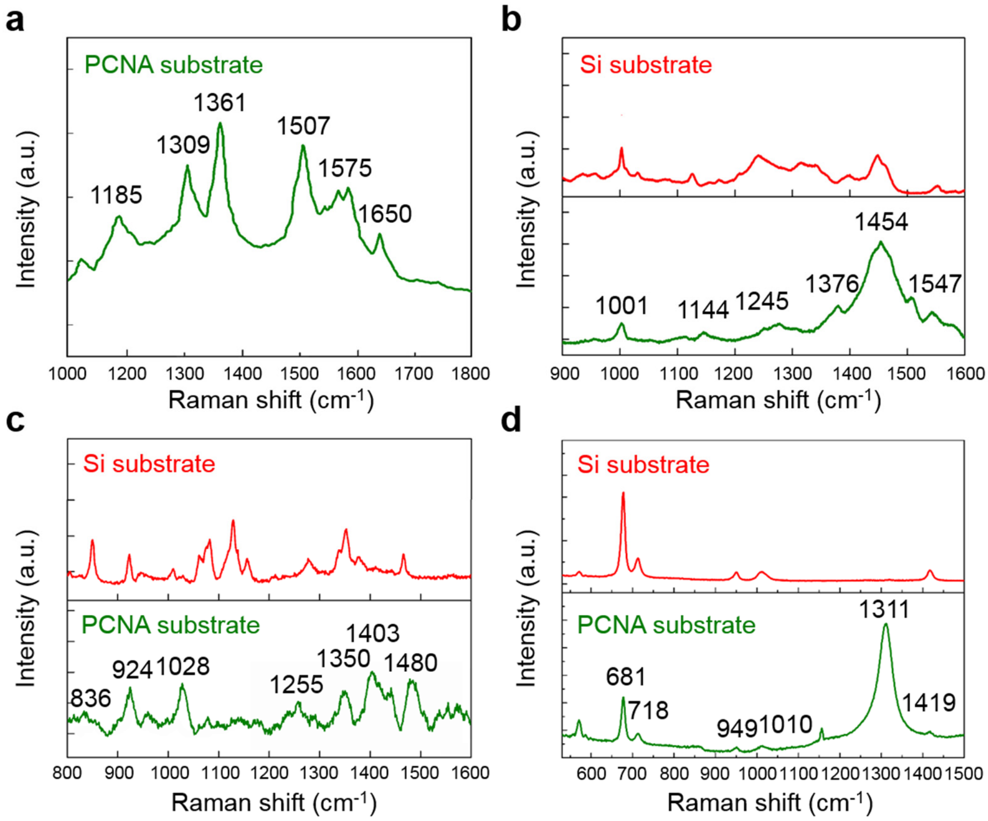

Supplementary Fig. 9 | Raman peak assignments of R6G, β-lactoglobulin, glucose, and DMSO. a, R6G. **b**, β-lactoglobulin. **c**, glucose. **d**, DMSO.

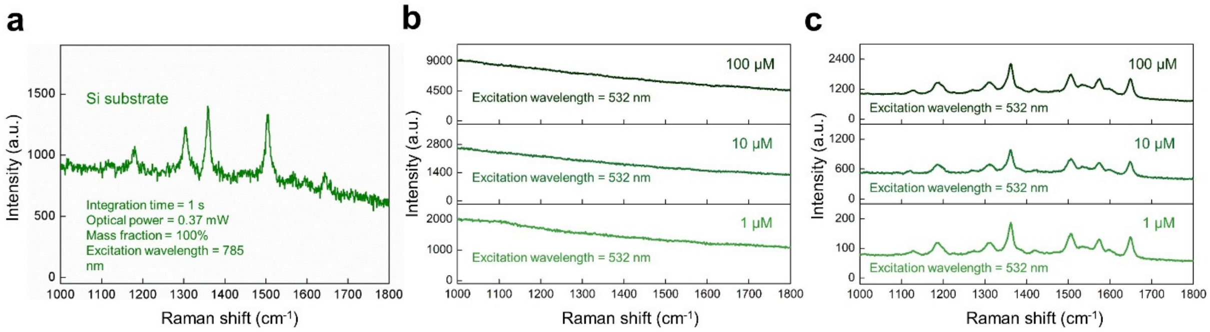

Supplementary Fig. 10 | Fluorescence quenching of the PCNA substrate. a, Raman spectrum of R6G on the silicon substrate at an excitation wavelength of 785 nm. **b,** Raman spectra of R6G at different concentrations on the silicon substrate for an integration time of 30 s with an excitation power and wavelength of 1 mW and 532 nm, respectively. **c,** Raman spectra of R6G at different concentrations adsorbed on the PCNA substrate for an integration time of 30 s with an excitation power and wavelength of 1 mW and 532 nm, respectively.

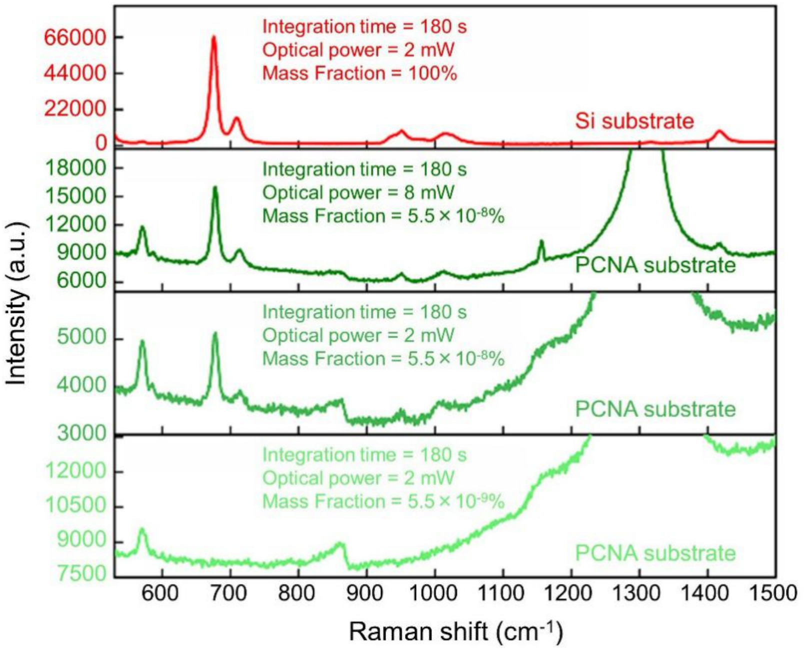

Supplementary Fig. 11 | Raman spectra of the DMSO on the silicon and PCNA substrates. Measured Raman spectra of the DMSO solutions with different mass fractions on the silicon and PCNA substrates. With the enhancement of the PCNA substrate, all the Raman peaks of the DMSO solutions were identifiable even at the very low mass fraction of 5.5×10^{-8}%.

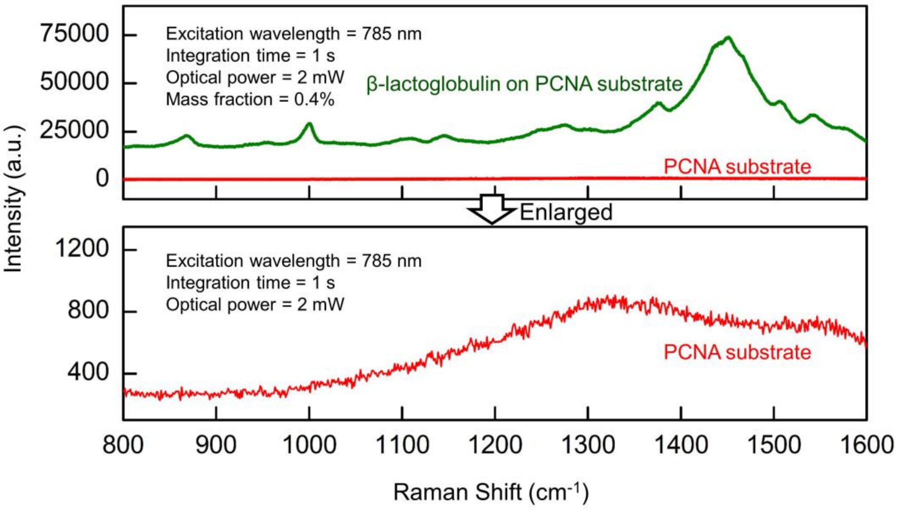

Supplementary Fig. 12 | Raman spectrum of the PCNA substrate. The Raman intensity of the PCNA substrate is a few orders of magnitude smaller than the enhanced Raman spectrum of β-lactoglobulin probed under the same conditions. Furthermore, its characteristic peaks are very broad and submerged by the enhanced Raman spectra of probed molecules as a broad background.

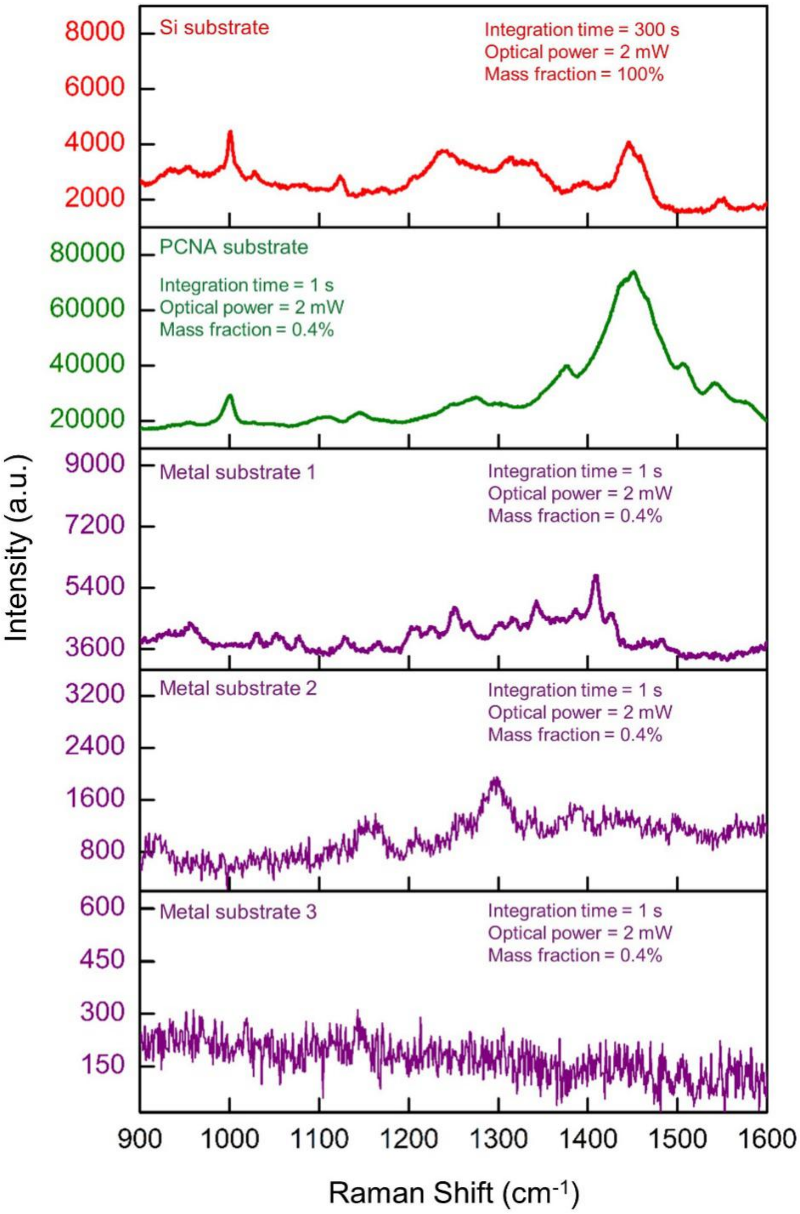

Supplementary Fig. 13 | Raman spectrum of β-lactoglobulin on different substrates. Metal substrates 1, 2, 3 are three types of commercial substrates: silver-gold hybrid substrate produced by SERSitive Co., silver substrate produced by ATO ID, and silver substrate produced by Labguide Co., respectively.

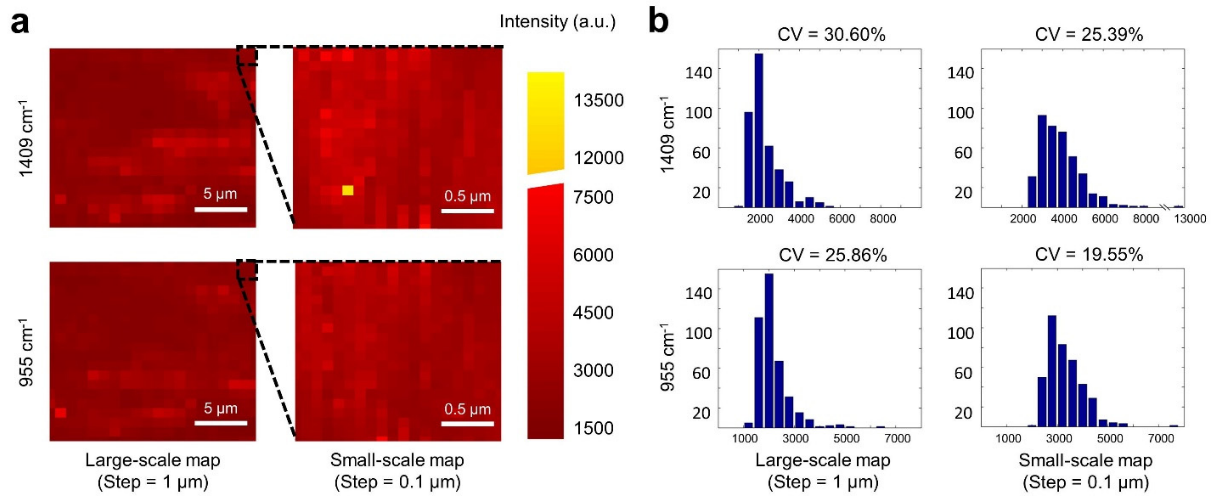

Supplementary Fig. 14 | SERS mapping of β-lactoglobulin on the commercial metal substrate. a, SERS maps on the metal substrate, showing high surface inhomogeneity in enhancement factor at two characteristic Raman shifts of β-lactoglobulin on both large and small scales with a step size of 1 μm and 0.1 μm, respectively. **b**, Histograms of the enhancement factors on the large and small scales.

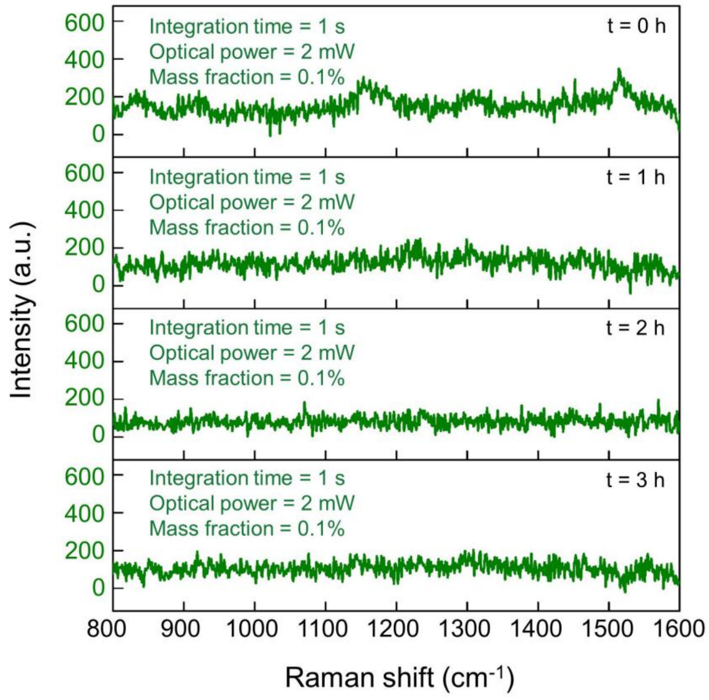

Supplementary Fig. 15 | Time-to-time fluctuations of the commercial metal substrate in the Raman spectrum. Except for the small Raman peaks at t = 0 h, there are almost no peaks in the Raman spectra at t = 1-3 h, showing poor reproducibility.

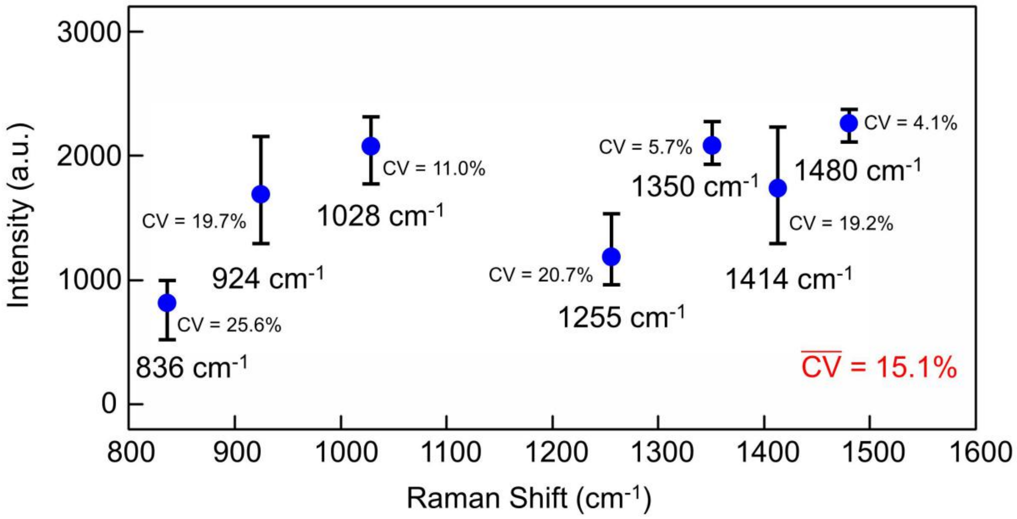

Supplementary Fig. 16 | Quantitative analysis of the temporal intensity fluctuations of the characteristic Raman peaks of glucose probed on the PCNA substrate. The average CV value within 4 hours is as small as 15.1% as opposed to most metal SERS substrates that cannot be used for more than 1 hour as they are easily oxidized in the air. The error bars show the temporal intensity fluctuation ranges of the Raman peaks shown in Fig. 3e. The blue dots show the mean values of the Raman peak intensity values measured at different times.

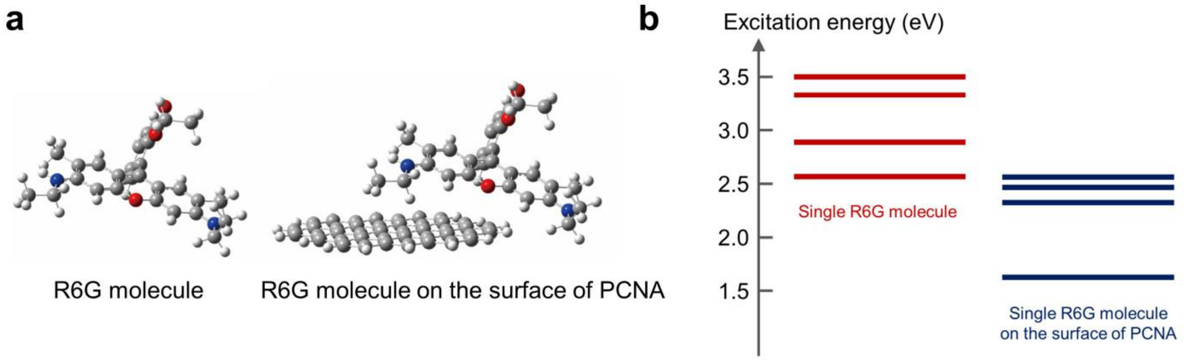

Supplementary Fig. 17 | Theoretical analysis of the chemical mechanism. a, Models used for the calculations. **b,** Excitation energy levels of a single R6G molecule and a single R6G molecule on the surface of the PCNA.

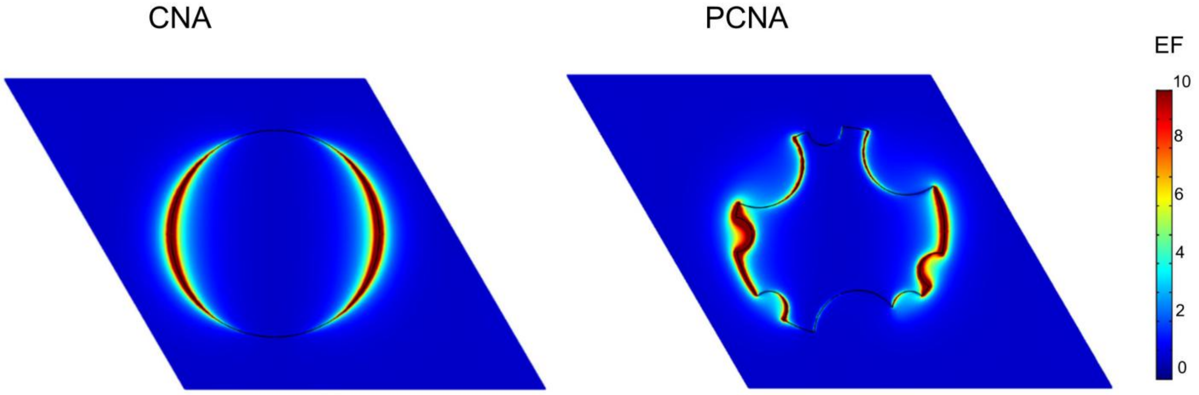

Supplementary Fig. 18 | Simulated electromagnetic contribution to the Raman enhancement factor (EF). As surface-enhanced Raman intensity is proportional to the fourth power of the localized electric-field magnitude, we calculated the average EM enhancement factor on the entire space by using $EF = \int |E(x,y,z)/E_0|^4 dv \,/ \int dv$, where $E(x,y,z)$ is the electric-field magnitude at the coordinate (x, y, z) in the near-field region; E_0 is the electric-field magnitude of the incident light. The integrated volume $\int dv$ is the volume of the unit cell that has excluded the volume of the nanowire. Our analysis results indicate that the average electromagnetic contributions to the enhancement factors on the CNA and PCNA substrates are found to be 1.6 and 1.8, respectively.

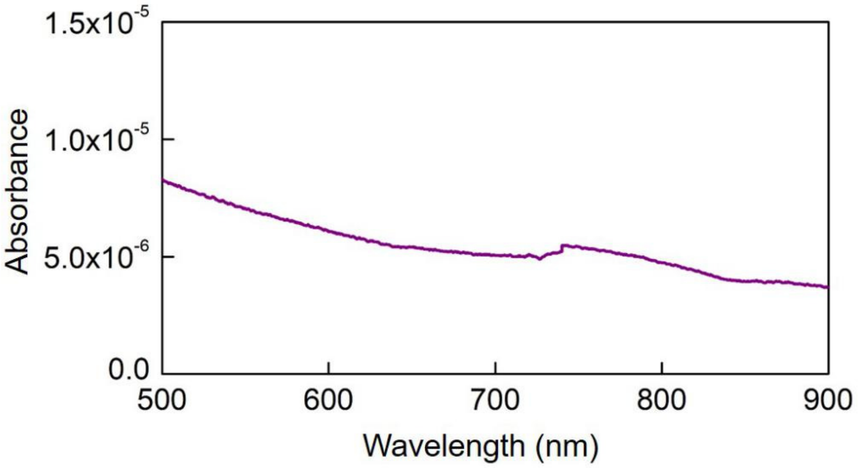

Supplementary Fig. 19 | Absorption spectrum of a β-lactoglobulin solution with a mass fraction of 0.4% and an optical path length of 30 μm. These values were deduced by Beer-Lambert law via measuring the β-lactoglobulin solution with a mass fraction of 0.4% and an optical path length of 1 cm. The absorbance of the molecule in this spectral region is very small.

Supplementary Table 1 | Comparison of different types of SERS substrates.

Material	Heat generation	Reproducibility	Biocompatibility	Enhancement factor	Ref.
**Metal (Ag)**	High	Very poor	Poor	10^9 at hot spots<br>10^5 on average	10
**Metal (Au)**	High	Very poor	Poor	10^9 at hot spots<br>10^5 on average	11
**Graphene**	Low	Poor	High	Less than 10^3	12-14
**Semiconductor (MoS_2)**	Low	Poor	Poor	10^5	15
**Semiconductor (Al_xWO_3)**	Low	High	Poor	10^4	16
**PCNA**	Low	Very high	High	10^6	This work

Supplementary Table 2 | Assignment of major peaks in the Raman spectra of measured samples.

Sample	Wavenumber (cm^{-1})	Assignment
R6G	1185	C–H bending
	1309	C–C stretching
	1361	C–C stretching
	1507	C–C stretching
	1575	C–C stretching
	1650	C–C stretching
β-lactoglobulin	1001	Phenylalanine
	1144	C–N stretching
	1245	Amide III (β-sheet)
	1376	C–H deformation, Tryptophan
	1454	CH_3 (asymmetric), CH_2, CH bending
	1547	Tryptophan
glucose	836	C–C stretching
	924	C–H bending
	1028	C–O stretching
	1255	CH_2 twisting
	1350	CH_2 wagging
	1403	CH_2 bending
	1480	CH_2 bending
DMSO	681	C–S stretching
	718	C–S stretching
	949	C–H bending
	1010	S=O stretching
	1311	C–H deformation
	1419	CH_3 deformation

Supplementary Table 3 | Estimated numbers of molecules of the measured samples in the probed volume.

Sample	Probed volume (μm^3)	Density (g/cm^3)	Mass fraction (%)	Molar concentration (M)	Number of molecules (mol)
β-lactoglobulin powder	1.5	1.25	100	6.8×10^{-2}	1.0×10^{-16}
β-lactoglobulin solution	1.5	1-1.25	0.4	2.7×10^{-4}	4.0×10^{-19}
glucose powder	1.5	1.54	100	8.7	1.3×10^{-14}
glucose solution	1.5	1-1.54	0.1	8.7×10^{-3}	1.3×10^{-17}
DMSO	1.5	1.1	100	14.1	2.1×10^{-14}
DMSO solution	1.5	1-1.1	5.5×10^{-8}	7.8×10^{-9}	1.2×10^{-23}

Supplementary References

1. Kim, T. W. *et al.* A synthetic route to ordered mesoporous carbon materials with graphitic pore walls. *Angew. Chem. Int. Ed.* **42**, 4375-4379 (2003).
2. Hildebrandt, P. *et al.* Surface-enhanced resonance Raman spectroscopy of Rhodamine 6G adsorbed on colloidal silver. *J. Phys. Chem.* **88**, 5935-5944 (1984).
3. Jung, Y. M. *et al.* Two-dimensional infrared, two-dimensional Raman, and two-dimensional infrared and Raman heterospectral correlation studies of secondary structure of β-lactoglobulin in buffer solutions. *J. Phys. Chem. B* **104**, 7812-7817 (2000).
4. Koenig, J. L. *et al.* Raman scattering of Chymotrypsinogen A, Ribonuclease, and Ovalbumin in the aqueous solution and solid state. *Biochemistry* **11**, 2505-2520 (1972).
5. Mathlouthi, M. *et al.* Laser-Raman spectra of D-glucose and sucrose in aqueous solution. *Carbohyd. Res.* **81**, 203-212 (1980).
6. Selvarajan, A. Raman spectrum of dimethyl sulfoxide (DMSO) and the influence of solvents. *Proc. Indian Acad. Sci.* **64**, 44-50 (1966).
7. Gorobets, M. I. *et al.* Raman study of solvation in solutions of lithium salts in dimethyl sulfoxide, propylene carbonate and dimethyl carbonate. *J. Mol. Liq.* **205**, 98-109 (2015).
8. Shikata, T. *et al.* Dimeric molecular association of dimethyl sulfoxide in solutions of nonpolar liquids. *J. Phys. Chem. A* **116**, 990-999 (2012).

9. McMeekin, T. L. *et al.* Partial specific volume of the protein and water in beta‐lactoglobulin crystals. *J. Polym. Sci.*, **12**, 309-315 (1954).
10. Camden J. P. *et al.* Probing the structure of single-molecule surface-enhanced Raman scattering hot spots. *J. Am. Chem. Soc*. **130**, 12616-12617 (2008).
11. Dasary, S. S. R. *et al.* Gold nanoparticle based label-free SERS probe for ultrasensitive and selective detection of trinitrotoluene. *J. Am. Chem. Soc.* **131**, 13806-13812 (2009).
12. Kang, L. *et al*. Recent progress in the applications of graphene in surface-enhanced Raman scattering and plasmon-induced catalytic reactions. *J. Mater. Chem. C* **3**, 9024-9037 (2015).
13. Huang, S. *et al.* Molecular selectivity of graphene-enhanced Raman scattering. *Nano Lett.* **155**, 2892-2901 (2015).
14. Feng, S. M. *et al.* Ultrasensitive molecular sensor using n-doped graphene through enhanced Raman scattering. *Sci. Adv.* **2**, e1600322 (2016).
15. Zheng, Z. H. *et al.* Semiconductor SERS enhancement enabled by oxygen incorporation. *Nat. Commun.* **8**, 1993 (2017).
16. Cong, S. *et al.* Electrochromic semiconductors as colorimetric SERS substrates with high reproducibility and renewability. *Nat. Commun.* **10**, 678 (2019).
