# Supplementary material for: Porous carbon nanowire array for surface-enhanced Raman spectroscopy
Source: Nat Commun. 2020 Sep 24;11:4772. doi: 10.1038/s41467-020-18590-7 (PMC7519110; doi:10.1038/s41467-020-18590-7)
Supplement: Supplementary file 2 — Peer Review File [file 41467_2020_18590_MOESM2_ESM.pdf]

**Editorial Note:** This manuscript has been previously reviewed at another journal that is not operating a transparent peer review scheme. This document only contains reviewer comments and rebuttal letters for versions considered at Nature Communications.
